# Simultaneous Silencing of Xylanase Genes in *Botrytis cinerea*

**DOI:** 10.3389/fpls.2017.02174

**Published:** 2017-12-22

**Authors:** Néstor García, Mario A. González, Celedonio González, Nélida Brito

**Affiliations:** Área de Bioquímica y Biología Molecular, Departamento de Bioquímica, Microbiología, Biología Celular y Genética, Sección de Biología, Universidad de La Laguna, San Cristóbal de La Laguna, Spain

**Keywords:** endo-xylanase, multiple gene silencing, RNAi, *Botrytis cinerea*, virulence

## Abstract

The endo-β-1,4-xylanase BcXyn11A is one of several plant cell-wall degrading enzymes that the phytopathogenic fungus *Botrytis cinerea* secretes during interaction with its hosts. In addition to its enzymatic activity, this protein also acts as an elicitor of the defense response in plants and has been identified as a virulence factor. In the present work, other four endoxylanase coding genes (*Bcxyn11B*, *Bcxyn11C*, *Bcxyn10A*, and *Bcxyn10B*) were identified in the *B. cinerea* genome and the expression of all five genes was analyzed by Q-RT- PCR *in vitro* and *in planta.* A cross-regulation between xylanase genes was identified analyzing their expression pattern in the Δ*Bcxyn11A* mutant strain and a putative BcXyn11A-dependt induction of *Bcxyn10B* gene was found. In addition, multiple knockdown strains were obtained for the five endoxylanase genes by transformation of *B. cinerea* with a chimeric DNA construct composed of 50-nt sequences from the target genes. The silencing of each xylanase gene was analyzed in axenic cultures and during infection and the results showed that the efficiency of the multiple silencing depends on the growth conditions and on the cross-regulation between them. Although the simultaneous silencing of the five genes was observed by Q-RT-PCR when the silenced strains were grown on medium supplemented with tomato extract, the endoxylanase activity measured in the supernatants was reduced only by 40%. Unexpectedly, the silenced strains overexpressed the *Bcxyn11A* and *Bcxyn11C* genes during the infection of tomato leaves, making difficult the analysis of the role of the endo-β-1,4-xylanases in the virulence of the fungus.

## Introduction

Endo-β-1,4-xylanases (E.C. 3.2.1.8) hydrolyze the β-(1,4)-xylosidic linkages between two xylosyl residues breaking the linear backbone of xylan (Pollet et al., 2010), the main hemicellulosic component of the plant cell wall. According to the Carbohydrate-Active Enzymes (CAZy) database, most xylanases belong to the glycosyl hydrolase families 10 (GH10) and 11 (GH11), although some enzymes have also been classified into families 5, 8, 16, 26, 30, 43, and 62 (Paës et al., 2012). GH10 xylanases are acidic proteins of about 30 kDa, while GH11 enzymes usually have lower molecular weights (∼22 kDa) and pI ranges between 2 and 11. GH10 members are also reported to be less substrate-specific (Pollet et al., 2010). The general structure of the GH10 xylanases catalytic domain is an eight-fold (β/α) barrel, with the active site located in a cleft (Natesh et al., 2003), while GH11 xylanases adopt a “β-jelly roll” folding that resembles the shape of a partially closed right hand, with the two catalytic glutamate residues on the concave side of the palm (Paës et al., 2012). In addition to the catalytic domain, these proteins frequently contain a carbohydrate-binding module (CBM) involved in binding to polysaccharides of the plant cell wall (Hervé et al., 2010). CBMs are also classified into more than 60 families in the CAZy database, based on their amino acid sequence similarity (Cantarel et al., 2009).

The *B. cinerea* GH11 xylanase BcXyn11A (Bcin03g00480) has been reported previously to act as an elicitor of the plant immune system and is required for full virulence regardless of its enzymatic activity (Brito et al., 2006; Noda et al., 2010). The *Bcxyn11A* gene deletion reduced only partially the total endoxylanase activity measured in the *B. cinerea* culture medium (Brito et al., 2006), probably as a result of the presence of multiple xylanase genes in the *B. cinerea* genome. Further investigation of the xylanase gene family in virulence would require the generation of multiple gene knockouts, but the limited number of resistance markers available for this fungus would make this a complex task and, therefore, new molecular approaches are required.

In this context, Post-Transcriptional Gene Silencing (PTGS) emerges as a powerful alternative. Since its discovery, it has been used for the targeted downregulation of genes in numerous fungal species (Armas-Tizapantzi and Montiel-González, 2016; Bashyal and Aggarwal, 2016), including *B. cinerea*. Sense-antisense (Schumacher et al., 2008; Patel et al., 2010; Giesbert et al., 2012; Espino et al., 2014) and stem-loop (Rolland and Bruel, 2008; Schumacher et al., 2008; Giesbert et al., 2012; Espino et al., 2014) strategies have been developed to generate specific double-stranded RNA (dsRNA) in *B. cinerea* cells. dsRNAs are processed by the enzymatic silencing machinery to produce small-interfering RNAs (siRNAs) that target their complementary mRNA, triggering gene silencing (Chang et al., 2012; Bashyal and Aggarwal, 2016). As a tool for genetic manipulation, PTGS has the advantage that it makes possible to co-silence multiple genes in a single transformation event (Salame et al., 2011; Quoc and Nakayashiki, 2015). In *Cladosporium fulvum*, the use of an inverted repeat chimeric sequence composed of the first exon from six hydrophobin coding genes as silencing trigger, caused downregulation of the six genes (Lacroix and Spanu, 2009). Following a similar approach, 10 endoxylanase (Nguyen et al., 2011) or 9 cellulase coding genes (van Vu et al., 2012) were successfully co-silenced in *Magnaporthe oryzae*. In *B. cinerea*, the co-silencing of *Bcsod1 and Bcass1* genes is the only case reported so far in which simultaneous gene silencing has been achieved (Patel et al., 2010). The vector used for the construction of *Bcsod1-*silencing plasmid contained a DNA fragment of *Bcass1* ORF instead of the *BctubA* terminator due to a previous miss-annotation of both genes, and its use caused the inadvertent *Bcass1* gene silencing (Patel et al., 2010).

In the present work, all GH10 and GH11 xylanase coding genes have been identified in *B. cinerea* genome and the expression profile of each of them has been studied in detail by Q-RT-PCR under different *in vitro* growth conditions and *in planta*. Knockdown strains have been generated by simultaneously silencing all xylanase coding genes, and phenotypical characterization of the knockdown strains was carried out to analyze the role of these hydrolases in fungal physiology and virulence.

## Materials and Methods

### Organisms and Growth Conditions

*Botrytis cinerea* B05.10 (Büttner et al., 1994) was used as wild type strain and a knockout mutant for *Bcxyn11A* gene (Brito et al., 2006; Noda et al., 2010) was used when indicated. All *B. cinerea* strains were kept as conidial suspensions in 15% (v/v) glycerol at -80°C for long storage, or were grown on 1% (w/v) malt extract (Oxoid) plates at 22°C for routine use. Conidia were prepared as described by Benito et al. (1998) from cultures on tomato-plates (25% (w/v) homogenized tomato fruits, 1.5% (w/v) agar, pH 5.5) and were routinely quantified at 600 nm with a spectrophotometer. The relationship between DO_600_ and number of conidia per ml was calculated for our spectrophotometer with a counting chamber. GB5 minimal media (0.3% (w/v) Gamborg’s B5, 10 mM KH_2_PO_4_ and 0.05% (v/v) Tween-80) was supplemented with 1% (w/v) beechwood xylan (Sigma-Aldrich) or 2% (w/v) glucose as indicated. To prepare liquid culture media containing plant extracts, a dialysis bag containing tomato or strawberry fruit extract was included in GB5 medium, as described previously (Espino et al., 2010). Media were supplemented with 100 μg/ml hygromycin and/or nourseothricin and 1.5% (w/v) agar, when required. *Escherichia coli* XL1-Blue, SURE-2 (Stratagene) and derived strains were routinely grown in LB [1% (w/v) Bacto tryptone, 0.5% (w/v) Yeast extract, 1% (w/v) NaCl pH 7.5], supplemented when necessary with 50 μg/ml ampicillin or 10 μg/ml tetracycline, and 1.5% (w/v) agar. *Solanum lycopersicum* cv. Moneymaker was maintained in a growth chamber at 22°C, 70% humidity, and with a light/dark cycle of 14 h light/10 h dark. Plants were watered three times per week, one of them including Universal Liquid Fertilizer (COMPO) as instructed by the manufacturer.

### Standard Molecular Techniques

For *E. coli* plasmid extraction, Quick Clean 5M Miniprep (GenScript) was used, following the manufacturer’s instructions, and only when high amount of plasmid DNA was needed, the method described by Sambrook and Russell (2001) was used. PCR amplifications were made with *Phusion* High-Fidelity DNA Polymerase (New England Biolabs) when the DNA product was to be used in cloning experiments, and *Taq* polymerase (GenScript) in any other case. All oligonucleotides used (Supplementary Table S1) were from Integrated DNA Technology. The purification of the PCR products was carried out with Quickclean PCR purification kit (Genscript) and purification of DNA fragments from agarose gels with Zymoclean Gel DNA Recovery kit (Zymo Research), following the manufacturer’s instructions. Ligation experiments were done with DNA Ligation Kit – Mighty Mix (Takara). Transformation of competent *Escherichia coli* cells was done as explained elsewhere (Inoue et al., 1990).

### Bioinformatics Analysis

To identify the *B. cinerea* GH10 and GH11 xylanase coding genes, a Blast (Altschul et al., 1997) search was carried out in the *B. cinerea* genome database^1^ (van Kan et al., 2016), using as queries amino acid sequences of well-characterized xylanases from other fungi (Supplementary Table S2) with known 3D structure, as well as BcXyn11A. Protein domains were searched against the Pfam database^2^ (Finn et al., 2016) and Prosite server^3^ (Sigrist et al., 2013). The new protein sequences were used as query sequences to perform a new Blast-P against the non-redundant protein sequences database at the National Centre for Biotechnology Information (NCBI). Multiple alignments of protein sequences were carried out with Clustal-Omega^4^ (Sievers et al., 2011) and the EMBOSS Stretcher program (Rice et al., 2000) was used for used for sequence similarity analysis^5^. Prediction of signal sequences for secretion was carried out with SignalP 4.1^6^ (Petersen et al., 2011) and *N-* or *O-*glycosylated sites were predicted with the NetNGlyc 1.0^7^ (Gupta et al., 2004) and NetOGlyc 4.0^8^ (Steentoft et al., 2013) servers, respectively. Theoretical physiochemical parameters such as molecular weight, isoelectric point and the aliphatic index of each protein were calculated on Expasy’s ProtParam server^9^ (Gasteiger et al., 2005).

### Generation of *Botrytis cinerea* Knockdown Strains

To generate knockdown strains for the xylanase coding genes identified in the *B. cinerea* genome, a chimeric sequence that would act as the silencing trigger was designed as follows. A region of 21-nt fulfilling the basic requirements to generate the corresponding siRNAs (Petri and Meister, 2013) was identified from each xylanase gene using the SVM RNAi server^10^, with the default parameters. In the case of the *Bcxyn10B* gene two regions with the same maximum score were selected. Both 5′ and 3′ ends of each region were then extended by 13–16 bp, so that a 50-bp region of each gene was selected (Supplementary Figure S1). The chimeric sequence of 300 bp generated by the fusion of these fragments was named Hom_Xyl (Supplementary Figure S1) and was completed with an additional 22-nt tail at its 3′-end, introducing *Sac*I, *Fse*I and *Pac*I restriction sites (Supplementary Figure S1). The resulting 322-pb fragment was chemically synthesized (GeneScript) and provided in the pUC57-6CXs plasmid.

The Hom_Xyl fragment was amplified from pUC57-6CXs by PCR with primers XYL-FW(*Nco*I)/XYL-RV(*Not*I+*BamH*I) or XYL-FW(*Not*I+*BamH*I)/XYL-RV(*Nco*I) (Table S1), generating similar 336-bp product, both with the Hom_Xyl sequence but with switched restriction sites at their 5′ and 3′ ends. Each PCR product was digested with *Nco*I and *Not*I and cloned into the pNDN-OGG or pNAH-OGG vector (Schumacher, 2012) respectively, generating pNDN-Xyl and pNAH-Xyl plasmids (Supplementary Figure S1). The plasmids pNDN-OGG and pNAH-OGG are designed for targeted integration of foreign DNA in the *B. cinerea BcniaD* or *BcniiA* genes respectively. pNDN-Xyl encodes a “sense” transcript of Hom_Xyl sequence under the control of *OliC* promoter from *Aspergillus nidulans* and the glucanase terminator (Tg*luc*) from *B. cinerea*, and confers nourseothricin resistance, with the expression and the resistance cassettes flanked by *BcniaD* sequences for targeted integration (Supplementary Figure S1). pNAH-Xyl codes for the “antisense” transcript of Hom_Xyl under the same regulatory sequences described for pNDN-Xyl and confers hygromycin resistance, with both cassettes flanked by *BcniiA* sequences (Supplementary Figure S1). A third plasmid, pNDN-Xyl-Tail, was obtained by cloning in the *Not*I and *Bam*HI sites of pNDN-Xyl vector the 347-bp fragment amplified from pUC57-6CXs with primer pair XYL-FW(*N*otI)/LINK-RV(*Bam*HI) (Supplementary Table S1). pNDN-Xyl-Tail would express two inverted repeats of the Hom_Xyl sequence separated by the multiple cloning site to produce a dsRNA-hairpin structure.

To obtain a *B. cinerea* strain expressing the anti-sense Hom_Xyl sequence (strain BcXyl-AS), *B. cinerea* B05.10 protoplasts were transformed with *Hind*III-linearized pNAH-Xyl as described elsewhere (Hamada et al., 1997; van Kan et al., 1997) and hygromycin resistant transformants were analyzed by PCRs to check for the right integration events at the *BcniiA* locus (Supplementary Figure S2). BcXyl-AS homokaryosis was confirmed by ensuring the absence of the wild type *BcniiA* gene by PCR, and the absence of ectopic integrations of recombinant DNA was checked by Southern-blot (Supplementary Figure S2), using a digoxigenin-labeled probe specific for the *OliC* promoter and the DIG-DNA Labeling and Detection kit (Roche). The strain BcXyl-AS was then transformed with *Xba*I-linearized pNDN-Xyl plasmid to generate strains expressing both the sense and antisense Hom_Xyl sequence, and nourseothricin/hygromycin resistant transformants were analyzed by PCR and Southern-blot as explained before, to check for the correct integration at the *BcniaD* locus, ensure homokaryosis and discard ectopic integrations (Supplementary Figure S2). Two transformants, BcXyl-DT1 and BcXyl-DT2, were selected for further assays. In spite of several attempts, no site-directed knockdown homokaryotic strains were obtained by transforming B05.10 protoplasts with *Xba*I-linearized pNDN-Xyl-Tail plasmid.

### Quantitative Real-Time PCR (Q-RT-PCR)

Total RNA from mycelium or *B. cinerea*-infected tomato plants was isolated with the RNeasy Plant Mini Kit (Qiagen), and cDNA was synthetized using the iScript cDNA Synthesis Kit (Bio-Rad). Q-RT-PCR reactions were performed with an iCycler iQ Real-Time PCR Detection System (Bio-Rad), iQ SYBR Green Supermix (Bio-Rad) and the primers listed in Supplementary Table S1 designed to be intron-spanning to avoid amplification from genomic DNA. The *B. cinerea actA* gene (Bcin16g02020) was used as an internal reference control. When necessary, contaminant genomic DNA was eliminated by treatment with RNase-free DNaseI (Roche). Relative mRNA levels were calculated by the ΔΔCt method from the mean of three independent determinations of the threshold cycle (Ct), and using in each case the indicated control sample as calibrator (Schmittgen and Livak, 2008). Deviation from the mean for each sample was calculated from the standard deviation (SD) in the ΔΔCt value using the expression 2^-(ΔΔCt±*SD*)^.

### Endo-Xylanase Activity

Fungal strains were grown in different media and at the indicated times, mycelia were removed by filtration and the xylanase activity was measured in the supernatants following a modified version of the DNS method reported by Bailey (1988). Reaction mixtures contained 125 μl of 1% (w/v) beechwood xylan in 50 mM citrate buffer, pH 5.3, and 25 μl of culture filtrates. After incubation at 30°C for 30 min, the reactions were stopped by the addition of 300 μl of DNS solution (43.83 mM dinitrosalicylic acid, 21.25 mM phenol, 3.96 mM sodium sulfite, 708 mM potassium sodium tartrate), boiled for 5 min and cooled to room temperature. The increase of absorbance was recorded at 540 nm and one unit of xylanase activity was expressed as the amount of enzyme required to produce 1 μmol of reducing sugar (xylose equivalent) per minute and gram of fresh mycelial weight (μmol × min^-1^ × g^-1^).

### SDS–PAGE and Zymograms

To detect xylanase activity in acrylamide/bisacrylamide gels, the protocol reported by Liao et al. (2015) was adapted as follows. Proteins contained in 750 μl of indicated culture filtrates were precipitated as reported elsewhere (Wessel and Flügge, 1984), resuspended in SDS–PAGE loading buffer and fractionated in a 10%T acrylamide/bisacrylamide gel containing 0.1% (w/v) beechwood xylan. Protein electrophoresis were carried out in a Bio-Rad (Hercules, CA, United States) Mini-PROTEAN 3 system, according to the manufacturer’s instructions and gels were immersed in a 2.5% (v/v) Triton X-100 solution (1 h at room temperature), rinsed with H_2_O, and incubated in 50 mM citrate buffer pH 5.4 (1 h at 25°C). Gels were then rinsed with H_2_O, immersed in 0.1% (w/v) congo red solution (15 min at RT) and then in 1 M NaCl (15 min at RT). When necessary, the two last steps were repeated until bands were clearly seen. Finally, gels were dipped in a 0.5% (v/v) acetic acid solution (5 min at RT) to stop the reaction.

### Phenotypic Characterization of Knockdown Strains

Conidia production was estimated in tomato-plates inoculated with agar plugs taken from the edge of actively growing colonies on MEA. Plates were incubated for 3 days in the dark, irradiated with near-UV light for 12 h, and 4 days later conidia were collected and quantified as explained above. The number of sclerotia was counted directly from similar plates incubated for 15 days in continuous darkness.

Pathogenicity tests were carried out on detached leaves of *S. lycopersicum*. Plant tissues were inoculated with 5 μl drops of TGGK (60 mM KH_2_PO_4_, 10 mM glycine, 0.01% (v/v) Tween 20, 0.1 M glucose) containing 2.5 × 10^5^ conidia/ml, or with agar plugs containing young mycelium. Infected plant material was incubated at 22°C under high humidity conditions on water-soaked filter paper in closed containers, and lesions at different time points were photographed. Quantitative results are presented as the growth rate of the lesion area, approximated to an ellipse using Fiji software (Schindelin et al., 2012), expressed as cm^2^/day.

### Statistical Data Processing

The software package SPSS 17.0 (SPSS Inc., Chicago, IL, United States) was used for the statistical analysis. The normal distribution of data was analyzed with the Kolmogorv–Sminrov test. When data showed a normal distribution, a *T*-Student test was performed to determine statistically significant differences, and the non-parametric Mann–Whitney test was applied when not. Statistically significant differences (*p* = 0.05) are indicated with an asterisk.

## Results

### The *Botrytis cinerea* Genome Encodes Two Family GH10 and Three Family GH11 Endo-β-1,4-xylanases

A Blast-P search of the *B. cinerea* B05.10 genome was carried out using as queries either BcXyn11A or well-characterized xylanase sequences from other fungi (Supplementary Table S2), with known 3D structure. This search allowed the identification of two xylanases of family GH10 (Bcin03g03480 and Bcin05g06020) and two additional members of family GH11 (Bcin15g01600 and Bcin12g00090), which were named BcXyn10A, BcXyn10B, BcXyn11B and BcXyn11C, respectively. BcXyn10A shares 68.7% amino acid sequence identity with xylanase XynF3 from *Aspergillus oryzae* (Q96VB6), whose gene expression is not repressed by glucose (Kimura et al., 2002), and BcXyn10B showed 55.5% identity with the endo-β-1,4-xylanase D from *Talaromyces funiculosus* (Q5ZNB1), a versatile xylanase able to hydrolyse CM cellulose (Furniss et al., 2005). BcXyn11B has a sequence identity of 61.6% with XYNI, one of the four xylanases described in *Trichoderma reesei* (P36218) which is regulated by the transcription factor Xyr-1 (Stricker et al., 2008), and BcXyn11C is close related with the endo-1,4-β-xylanase B of *Phanerochaete chrysosporium* (B7SIW1, 56.5% sequence identity) that possess a type I carbohydrate-binding domain (Decelle et al., 2004).

**FIGURE 1:**
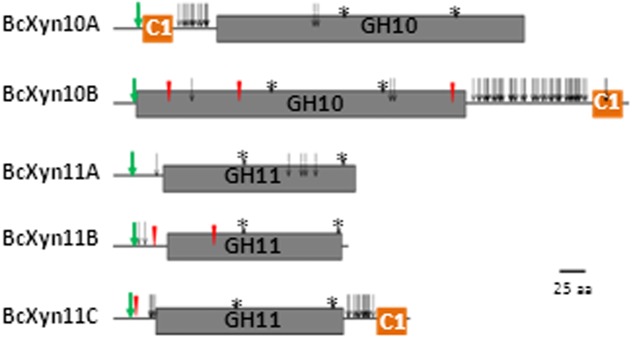
Structural features of the five xylanases coded by *B. cinerea* genome. Catalytic GH10 (Pfam PF00331) and GH11 (Pfam PF00457) domains and carbohydrate binding module 1 (C1, PF00734) are drawn to represent their relative positions along the protein chains of family GH10 and family GH11 xylanases. Predicted *N*-glycosylated and *O*-glycosylated residues are represented as red triangles and open arrows, respectively. Conserved glutamic acid residues of the catalytic domain are represented with asterisks and the signal peptide cut site as green arrows, respectively.

*In silico* analysis of the five protein sequences revealed the conserved GH10 and GH11 catalytic domains and a family 1 carbohydrate binding module (CBM1) in BcXyn10A, BcXyn10B and BcXyn11C linked to the catalytic domain with a hinge region rich in serine and threonine residues (**Figure 1**). A typical signal sequence was identified in the five enzymes (**Figure 1** and **Table 1**). BcXyn10B, BcXyn11B, and BcXyn11C were predicted to be *N*-glycosylated, and BcXyn10A, BcXyn10B and BcXyn11C were predicted to be heavily *O*-glycosylated in the linker region connecting the catalytic domain and the carbohydrate binding domain (**Figure 1** and **Table 1**). On the other hand, the ProtParam Server was used to compute some important physiochemical parameters which are recorded in **Table 1**.

**Table 1:** Bioinformatics analysis of family GH10 and GH11 xylanases encoded by *B. cinerea* genome.

Protein	SP^a^	aa^b^	MW (Da)^c^	pI^d^	AI^e^	N^f^	O^g^	CBM1
BcXyn10A	22–23	366	38675	7.13	76.83	0	18	+
BcXyn10B	17–18	470	49452	4.88	82.43	3	48	+
BcXyn11A	19–20	208	22046	8.04	59.09	0	5	-
BcXyn11B	19–20	203	21789	4.51	70.99	2	2	-
BcXyn11C	19–20	262	27355	5.76	43.93	1	19	+


*All physiochemical parameters were computed with ProtParam for the predicted mature sequences proteins (without signal peptide); signal peptide cutting position was predicted with SignalP server; *N*- or *O*-glycosylated sites were predicted with the NetNGlyc 1.0 and NetOGlyc 4.0 servers, respectively; CBM1 domain was identified by Pfam search. ^a^Amino acid residues involved in the cleavage of the signal sequence in the inmature protein. ^b^Amino acid residue number. ^c^Molecular weight (daltons). ^d^Isoelectric point. ^e^Alifatic index. ^f^Number of *N*-glycosilated residues. ^g^Number of *O*-glycosilated residues.*

The two *B. cinerea* GH10 xylanases share a relative low sequence identity (28.3%) between them, and the identity raised only slightly (to 32%) when just the catalytic domains were aligned. The pairwise alignments of GH11 xylanases showed that BcXyn11A and BcXyn11C had the highest similarity (48.6%) and BcXyn11B and BcXyn11C had the lowest (33.1%) and, again, the removal of BcXyn11C CBM1 from the alignment did not produce a significant increase in the identity percentage (57.5% for BcXyn11A and BcXyn11C, and 38.8% for BcXyn11B and BcXyn11C).

### The Five *B. cinerea* Xylanase Coding Genes Were Differentially Expressed in Axenic Culture and *in Planta*

**FIGURE 2:**
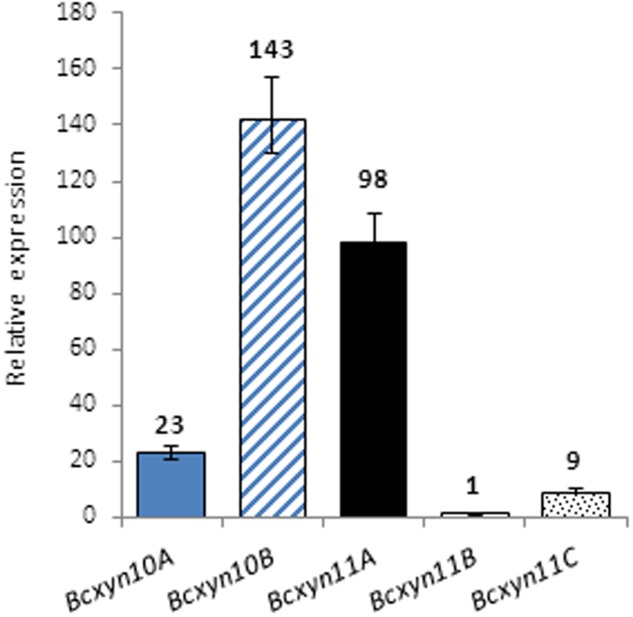
Expression of *B. cinerea* xylanase coding genes in ungerminated conidia. Total RNA was isolated from ungerminated conidia of B05.10 strain and the expression level of each xylanase gene was determined by Q-RT-PCR. *actA* gene was used as an internal reference control and relative expression was estimated as fold changes with respect to the expression level of *Bcxyn11B* gene, which was considered 1. Results are shown as mean ± SD for three technical replicates.

The expression of the five endoxylanase coding genes was analyzed by Q-RT-PCR. In ungerminated conidia, the five genes were expressed and *Bcxyn11B* mRNA was the least abundant (**Figure 2**), so that its expression level was used to calculate the relative expression of the other four in ungerminated conidia. The *Bcxyn10B* and *Bcxyn11A* transcripts were the most abundant (52.2 and 35.7%, respectively, of the total relative abundance) and, on the whole, relative abundance of family GH10 transcripts was slightly higher than that of family GH11 (**Figure 2**).

**FIGURE 3:**
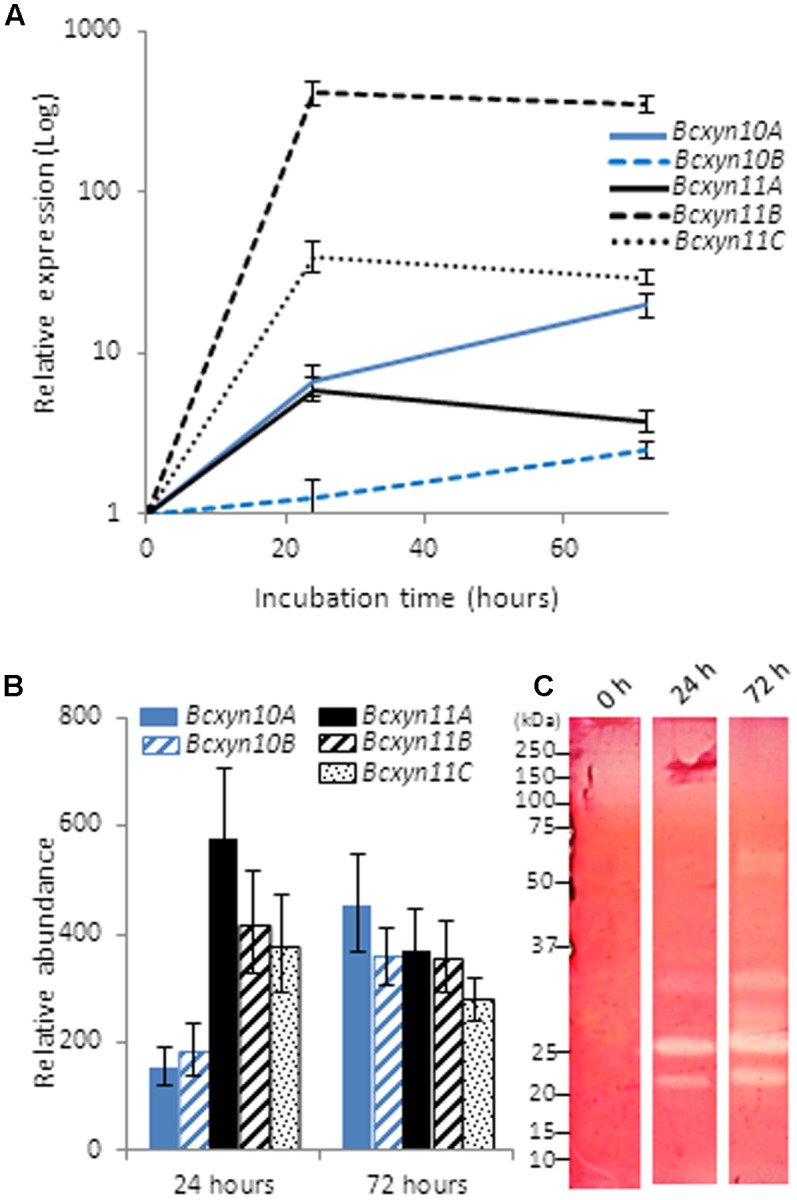
Xylan effect on the expression of *B. cinerea* xylanase coding genes. **(A)** Conidia from B05.10 strain were grown in GB5 supplemented with 1% beechwood xylan and total RNA was isolated after 24 and 72 h post inoculation. The expression level of each gene was estimated by Q-RT-PCR, using *actA* gene as an internal reference control. In this case, the relative expression was estimated as fold changes with respect to the expression level of each gene in ungerminated conidia. **(B)** The relative abundance of each transcript was calculated considering the expression levels of each gene in ungerminated conidia, and the fold changes in their expression after fungal growth in xylan, measured in **(A)**. Two independent biological replicates were performed and results are shown as mean ± SD for three technical replicates for each condition. **(C)** Secreted proteins in A were concentrated by precipitation with methanol/chloroform (Wessel and Flügge, 1984) and xylanases were detected on zymograms after SDS–PAGE (Laemmli, 1970), where 1% xylan was incorporated in the polyacrylamide gel.

The use of xylan as carbon source induced the upregulation of the five genes, but the induction pattern was different for each xylanase family (**Figure 3A**). The expression of GH11 genes increased after 24 h of growth and decreased slightly after 72 h, while both transcripts from GH10 increased almost linearly (**Figure 3A**). Interestingly, the least expressed genes in ungerminated conidia (*Bcxyn11B* and *Bcxyn11C*) were the most upregulated (up to 415 or 39-fold increase, respectively), while *BcXyn10B*, the most abundant transcript in ungerminated conidia, was only induced up to 1.2 times. As result, transcripts of family GH11 were relatively more abundant during the first hours of growth, while after 3 days the relative abundance of the five mRNAs was almost the same (**Figure 3B**). The levels of mRNAs did correlate with the endoxylanase activity detected in the fungal secretome, analyzed by SDS–PAGE and zymogram (**Figure 3C**).

**FIGURE 4:**
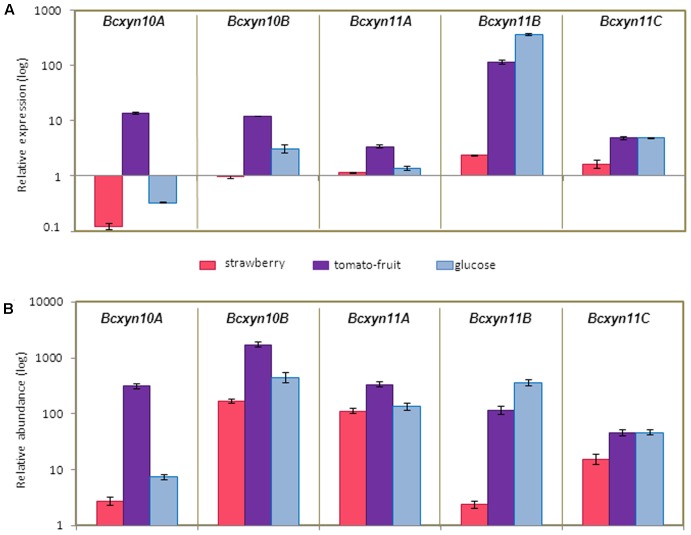
Expression of *B. cinerea* xylanase coding genes in different axenic cultures. **(A)** Conidia of B05.10 strain were germinated in GB5 supplemented with glucose or containing a dialysis bag enclosing strawberry or tomato-fruit extract, and xylanase mRNA levels were measured by Q-RT-PCR after 24 h at 20°C. *actA* gene was used as an internal reference control and the relative expression was estimated as fold changes with respect to the expression level of each gene in ungerminated conidia. Two independent biological replicates were performed and results are shown as mean ± SD for three technical replicates for each condition. **(B)** Relative abundance of xylanase mRNAs estimated in mycelia samples from **(A)**. Relative abundances were calculated considering the expression level of each gene in ungerminated conidia (**Figure 2**), and the fold changes in its expression after fungal growth in each culture medium, measured in **(A)**. Two independent biological replicates were analyzed and results are shown as mean ± SD for three technical replicates.

Different induction patterns were observed when the culture medium was supplement with different plant extracts (**Figure 4**). The use of strawberry extract produced a slight increase in the expression of *Bc*xy*n11B* and *Bcxyn11C*, while *Bcxyn10A* was downregulated and *BcXyn10B* and *Bcxyn11A* mRNA levels remained relatively constant (**Figure 4**). Tomato-fruit extract induced the expression of the five xylanase-coding genes (**Figure 4A**), and in spite of being *Bcxyn11B* gene the most upregulated (a 115-fold increase relative to ungerminated conidia), *Bcxyn10B* transcript showed the highest relative abundance after 24 h of growth (**Figure 4B**).

The carbon catabolite repression of the five xylanase genes was studied using glucose as carbon source (**Figure 4**) and the results showed that only *Bcxyn10A* was subject to catabolite repression (**Figure 4A**). Unexpectedly, the expression of *Bcxyn10B*, *Bcxyn11B* and *Bcxyn11C* increased, being the *Bcxyn10B* and *Bcxyn11B* mRNAs the most abundant transcripts after 24 h of growth on glucose (**Figure 4B**).

Taken together, these results show that the five xylanases are differentially expressed in axenic culture (**Figures 3**, **4**), with *Bcxyn11B* being the most upregulated gene as compared with ungerminated conidia in all condition tested, although its transcript never became the most abundant among the five genes (**Figures 3**, **4**). The two others members of the GH11 family, *Bcxyn11A* and *Bcxyn11C*, were the ones most prone to induction by xylan (**Figures 3A**, **4A**), and family GH10 genes were best upregulated when conidia were grown in tomato extract medium (**Figures 3**, **4**).

**FIGURE 5:**
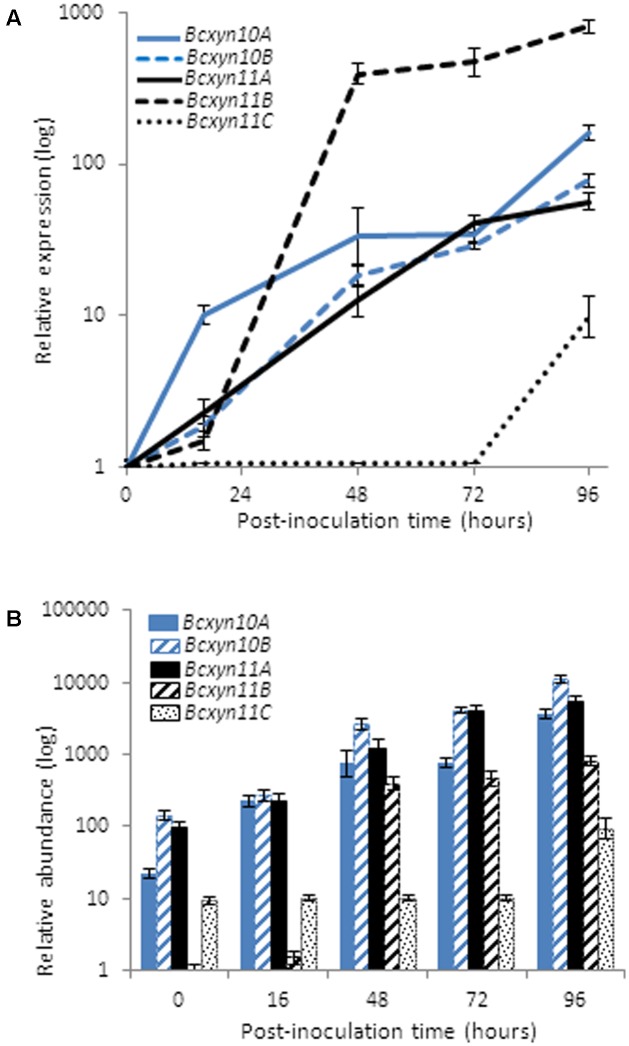
Expression of *B. cinerea* xylanase coding genes *in planta*. **(A)** Tomato leaves were inoculated with conidia of B05.10 strain and xylanase mRNA levels were measured by Q-RT-PCR through the infection process at the indicated times. *actA* gene was used as an internal reference control and the relative expression was estimated as fold changes with respect to the expression level of each gene in ungerminated conidia. Two independent biological replicates were performed and results are shown as mean ± SD for three technical replicates for each condition. **(B)** Relative abundance of xylanase mRNAs estimated in mycelia samples from A. Relative abundances were calculated considering the expression level of each gene in ungerminated conidia (**Figure 2**), and the fold changes in its expression after leaves inoculation at the indicated times, measured in **(A)**.

To gain insights on the putative role of each endo-β-1,4-xylanase in the fungus–plant interaction, their expression was analyzed by RT-PCR over the time course of the infection of tomato leaves with the *B. cinerea* wild type strain B05.10 (**Figure 5**). The results showed that all genes, except *Bcxyn11C*, were rapidly induced after inoculation and continued increasing for up to 96 h. mRNA levels increased by more than 800-fold in the case of *Bcxyn11B* (**Figure 5**). On the contrary, the expression of *Bcxyn11C* increased only at later stages of infection (**Figure 5A**). At 16 h after inoculation, both family GH10 and *Bcxyn11A* transcripts were the most abundant and continued increasing throughout the infection process (**Figure 5B**). On the other hand, the *Bcxyn11B* mRNA was the least abundant during the first hours of growth, but its levels increased after 48 h, reaching expression values similar to those of *Bcxyn10A, Bcxyn10B* and *Bcxyn11A* mRNAs (**Figure 5B**).

### The Expression of the Five *B. cinerea* Xylanase Coding Genes Is Co-regulated

**FIGURE 6:**
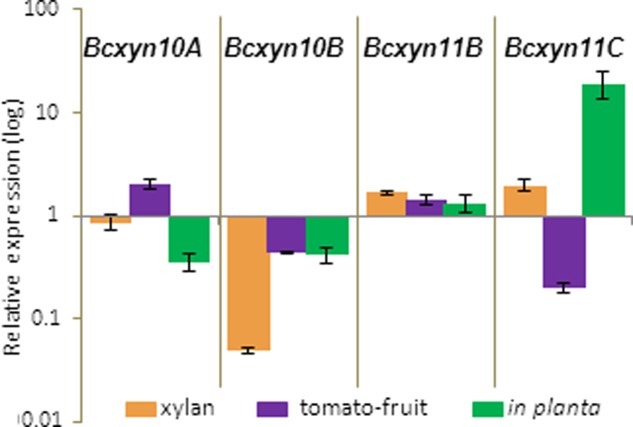
Relative expression of *Bcxyn10A*, *Bcxyn10B*, *Bcxyn11B* and *Bcxyn11C* genes in Δ*Bcxyn11A* strain. Conidia of Δ*Bcxyn11A* strain were grown in GB5 supplemented with 1% beechwood xylan for 72 h (xylan), containing a dialysis bag enclosing tomato fruit extract for 24 h (tomato-fruit) or were used to infect tomato leaves for 72 h (*in planta*). Xylanase mRNA levels were measured by Q-RT-PCR. *actA* gene was used as an internal reference control and the relative expression was estimated as fold changes with respect to the expression level of each gene in B05.10 strain grown in similar conditions. Two independent biological replicates were performed and results are shown as mean ± SD for three technical replicates for each condition.

The *Bcxyn11A* knockout mutant (Brito et al., 2006) was used to study the hypothetical co-regulation of the five *B. cinerea* xylanase-coding genes. The Δ*Bcxyn11A* strain was grown in minimal media supplemented with beechwood xylan or tomato extract, or was also used to inoculate tomato leaves, in order to measure the expression of the other four xylanase coding genes by Q-RT-PCR. mRNA levels are presented as fold increase or decrease relative to the expression level measured for the wild type strain grown under the same conditions (**Figure 6**).

With xylan as carbon source, the expression of the other two family GH11 genes were about two times higher in the mutant compared with strain B05.10, while the expression of *Bcxyn10B* was lower (**Figure 6**). However, when the Δ*Bcxyn11A* strain was grown in tomato extract medium, *Bcxyn10A* and *Bcxyn11B* were overexpressed about two-fold, while *Bcxyn10B* and *Bcxyn11C* were downregulated (**Figure 6**). During the infection of tomato leaves by the mutant, the expression of the xylanase coding genes was also altered. The transcript levels of *Bcxyn11B* and *Bcxyn11C* were higher in the Δ*Bcxyn11A* strain (up to 20-fold for *Bcxyn11C*) and a repression of both GH10 xylanase coding genes was observed (**Figure 6**).

### Simultaneous Silencing of Xylanase Coding Genes Occurs with Variable Efficiencies in *Botrytis cinerea*

In order to analyze the overall contribution of GH10 and GH11 xylanases to *B. cinerea* growth and virulence, knockdown strains were generated simultaneously for the five xylanase coding genes. First of all, the chimeric gene Hom_Xyl was designed containing a 50-nt fragment from each endoxylanase coding gene, except for *Bcxyn10B* for which two fragments were selected (Supplementary Figure S1). Each fragment included 21-nt predicted as the region with the maximum score to generate specific siRNAs for each xylanase gene by the SVMRNAi2.0 server. To check the putative off target silencing effect of Hom_Xyl, the 322-nt sequence was used as query in a BLAST search against the *B. cinerea* genome and no significant similarity with other DNA sequences was observed. Three silencing plasmids (pNDN-Xyl, pNAH-Xyl and pNDN-Xyl-Tail) were generated containing the chimeric gene Homo_Xyl (Supplementary Figure S1).

pNAH-Xyl was used to transform protoplasts of the wild type strain B05.10 resulting in a strain (BcXyl-AS) containing a single copy of the “anti-sense” Homo_Xyl expression cassette integrated at the *BcniiA* locus (Supplementary Figure S2). The BcXyL-DT1 and BcXyl-DT2 strains derive from BcXyl-AS by transformation with pNDN-Xyl plasmid, and harbor also a single copy of the “sense” Homo_Xyl expression cassette integrated at the *BcniaD* locus (Supplementary Figure S2). Finally, the pNDN-Xyl-Tail plasmid was used to transform strain B05.10 in order to express the construction designed and to generate a hairpin double-stranded RNA with the Homo_Xyl sequence, but non *BcniaD*-homokaryotic integrated transformants were obtained.

**FIGURE 7:**
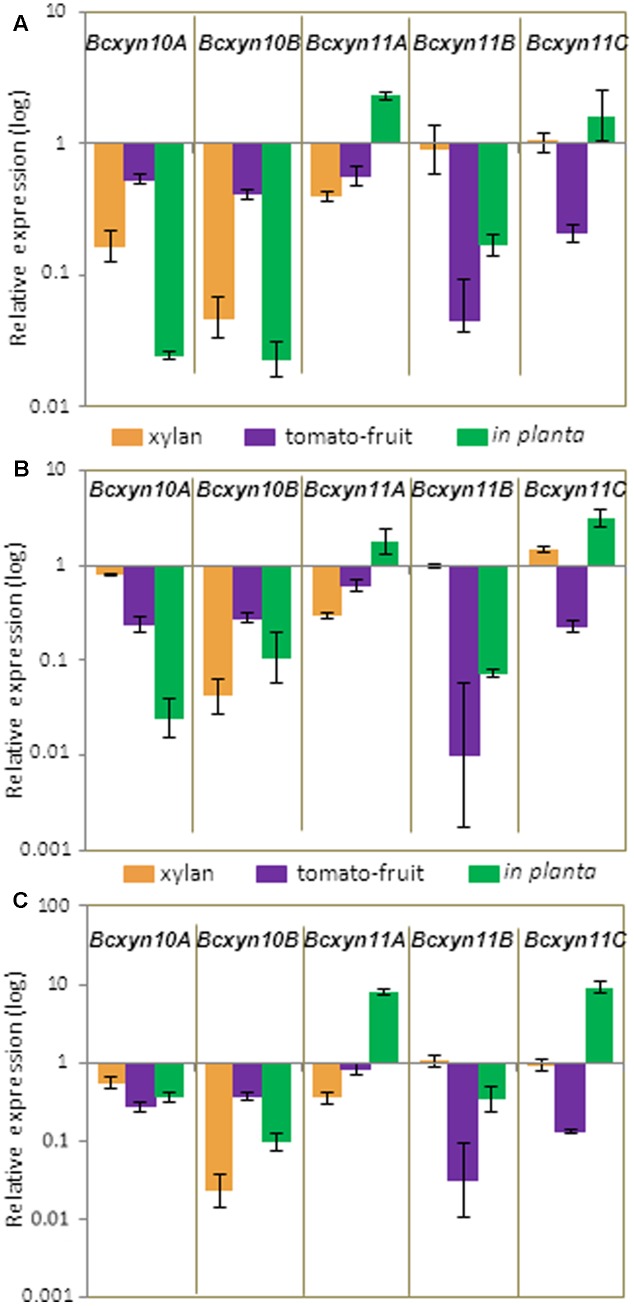
Relative expression of the five xylanase coding genes in the knockdown strains. Conidia of BcXyl-AS **(A)**, BcXyl-DT1 **(B)**, and BcXyl-DT2 **(C)** strains were grown in GB5 supplemented with 1% beechwood xylan for 72 h (xylan), containing a dialysis bag enclosing tomato fruit extract for 24 h (tomato-fruit) or were used to infect tomato leaves for 72 h (*in planta*), and xylanase mRNA levels were measured by Q-RT-PCR. Two independent biological replicates were performed and results are shown as mean ± SD for three technical replicates for each condition. *actA* gene was used as an internal reference control, and the relative expression was estimated as fold changes with respect to the expression level of each gene in B05.10 strain grown in similar conditions.

The strains BcXyl-AS, BcXyl-DT1, BcXyl-DT2 and B05.10 were grown under different conditions and the mRNA levels for each xylanase coding gene were measured by Q-RT-PCR. A similar downregulation pattern was observed for the five genes in the three transformants (**Figure 7**). After 24 h of growth in media supplemented with tomato extract, the five genes were simultaneously silenced, and a 95-75% reduction in transcripts levels was observed for *Bcxyn11B* and *Bcxyn11C* in all silenced strains (**Figure 7**). However, when the knockdown strains were grown in xylan, only three out of five xylanase coding genes were co-silenced (**Figure 7**). Family 10 xylanases and *Bcxyn11A* genes were downregulated (∼95% in the case of *Bcxyn10B*), while the expression of *Bcxyn11B* and *Bcxyn11C* remained similar to the wild type levels (**Figure 7**).

To analyze if the interaction with the host could also induce changes in the silencing pattern of the five genes, tomato leaves were inoculated with conidia from the four *B. cinerea* strains and the transcript levels of all xylanase genes were determined at 72 h after inoculation. Again, the expression of the two GH10 genes was reduced in the three knockdown mutants to almost to 10–20% of wild type levels, and relative abundance of *Bcxyn11B* mRNA was also reduced to 35%. Surprisingly, *Bcxyn11A* and *Bcxyn11C* were overexpressed (**Figure 7**).

**FIGURE 8:**
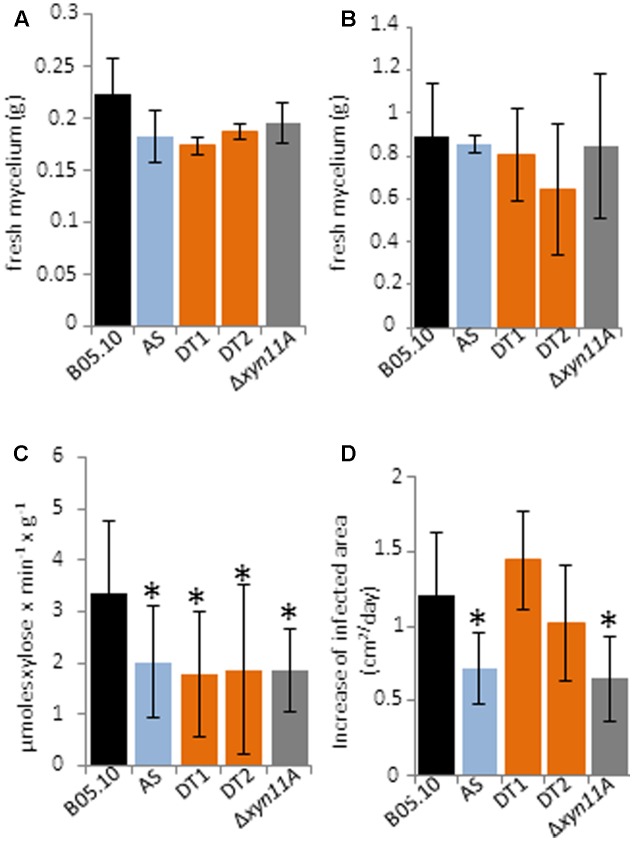
Phenotypic characterization of the knockdown strains. Conidia from the of BcXyl-AS (AS), BcXyl-DT1 (DT1), BcXyl-DT2 (DT2), Δ*Bcxyn11A* mutant (Δxyn11A) and wild type (B05.10) strains were used to inoculate GB5 supplemented with 1% beechwood xylan **(A)** or containing a dialysis bag enclosing tomato fruit extract **(B)**, at a final concentration of 10^6^ conidia/ml. After 72 or 24 h, respectively, mycelia were collected by filtration and weighted. Mean ± SD is shown for at least three biological replicates. **(C)** The fungal cultures grown as in **(B)** were filtered, and xylanase activity was measured in the culture medium by the DNS method with 1% beechwood xylan as substrate. One enzymatic unit is expressed as the amount of enzyme required to produce 1 μmol of reducing sugar (xylose equivalent) per minute and gram of fresh mycelial weight (Mean ± SD, *n* = 3). **(D)** Increase of growth rate of the infected area in tomato leaves inoculated with conidia of indicated strains as in **(A)** (mean ± SD; *n* ≥ 24).

The knockdown strains showed no changes in hyphal morphology, production of conidia and sclerotia or growth rate in tomato fruit extract plates (data not shown). Only a slight difference, without statistical significance, was observed for mycelium fresh weights between the knockdown and the wild type strains when xylan was used a carbon source (**Figure 8A**), while fresh weights were similar in liquid medium containing a dialysis bag enclosing tomato-fruit (**Figure 8B**). Endoxylanase activity was also measured in the culture filtrates and a reduction of about 40% was observed for the three knockdown strains when compared with B05.10 (**Figure 8C**). Unexpectedly, silencing of the five xylanase genes caused about the same decrease in the level of extracellular endo-β-1,4-xylanase activity as the deletion of *Bcxyn11A* (**Figure 8C**).

The ability of the knockdown strains to infect tomato leaves was also analyzed. The percentage of inoculations that were able to generate an expanding infection was similar in all strains, always above 90%. A delayed expansion of the infections from the primary necrotic lesions in tomato leaves was only observed for BcXyl-AS, but not for the other two knockdown strains (**Figure 8D**). The knock-out mutant Δ*Bcxyn11A* was also included in the pathogenicity tests, and the previously reported reduced virulence (Brito et al., 2006) was corroborated (**Figure 8D**).

## Discussion

### Endo-β-1,4-xylanases Are Redundant Enzymes in *Botrytis cinerea*

*Botrytis cinerea* secretes at least one endo-β-1,4 xylanase, BcXyn11A, involved in the degradation of xylan and in the induction of the hypersensitivity response in plants (Brito et al., 2006; Espino et al., 2010; Noda et al., 2010). In the present work, a Blast-P search (Altschul et al., 1997) of the *B. cinerea* B05.10 genome database allowed the identification of four additional endo-β-1,4-xylanases, two GH10 enzymes (BcXyn10A and BcXyn10B) and two GH11 enzymes (BcXyn11B and BcXyn11C), corroborating the results described by Amselem et al. (2011) for *B. cinerea* T4. Molecular weights and pIs of mature xylanases showed the expected values according to the family which they belong to (Motta et al., 2013). For both *B. cinerea* GH10 xylanases and BcXyn11C, a type-1 carbohydrate-binding module was predicted, besides the catalytic domain (**Figure 1**). The CBM1 motifs encompass 32–36 residues, four of which are conserved cysteines (Cantarel et al., 2009) and although it is described as the most abundant carbohydrate binding motif in the CAZymes of *B. cinerea* (Amselem et al., 2011), it is not often found in fungal GH11 xylanases (Paës et al., 2012). This domain targets the enzymes to the cellulose surface, but it might also help to indirectly localize the xylan substrate, increasing the catalytic efficiency (Paës et al., 2012), and contributing to the synergistic hydrolysis of the cell wall polysaccharides (Inoue et al., 2015).

The same degree of redundancy for GH10 and GH11 xylanases was described for *Sclerotinia sclerotiorum* (Amselem et al., 2011), a taxonomically related necrotrophic fungus, but also for the saprophytic fungus *Aspergillus oryzae* and for the hemi-biotroph *Gibberella zeae* (Amselem et al., 2011), indicating a no clear relationship between the number of hemicellulose-degrading enzymes and the lifestyle of these plant pathogens. Neither, differences in hemicellulose composition between cell walls of monocot and dicotyledonous plant species have been co-related with the arsenal of hemicellulases secreted by their pathogens (Kubicek et al., 2014; Zhao et al., 2014). It has been suggested that *B. cinerea* mainly used this set of enzymes for decomposition of plant cell walls, rather than for realizing nutrients (Amselem et al., 2011), but it would be interesting to verify their putative role as elicitors of the plant defenses as it has been described for BcXyn11A (Noda et al., 2010), as well as for other fungal xylanases (Bailey et al., 1990; Enkerli et al., 1999; Sella et al., 2013; Tundo et al., 2015).

### Family GH10 and Family GH11 Xylanases Are Differentially Expressed during Fungal Growth and Infection

Q-RT-PCR was used to analyze the expression of the whole set of endo-β-1,4-xylanases identified in the *B. cinerea* genome. The five genes were found to be expressed in ungerminated conidia (**Figure 2**), suggesting that the five enzymes might be involved in the early stages of fungal development. All of them were upregulated after the first 24 h of growth in xylan (**Figure 3**) or in tomato-fruit extract (**Figure 4**), although in strawberry extract only a slight upregulation of family GH11-coding genes was observed (**Figure 4**). Interestingly, although the *Bcxyn11B* gene was always the most overexpressed relative to ungerminated conidia in all condition tested, GH10 mRNAs exhibited the highest relative abundance in all growth conditions, except after 24 h of growth in xylan where GH11 transcripts were more abundant (**Figures 3**, **4**). Unexpectedly, *Bcxyn10A* was the only one of the five genes subjected to carbon catabolite repression (**Figure 4**). In previous work, we have experimentally identified both GH10 enzymes in the culture filtrate of *B. cinerea* grown in rich medium (González et al., 2014) and Fernández-Acero et al. (2010) also found both enzymes and BcXyn11C after fungal growth in medium supplemented with tomato cell walls. However, only BcXyn10B was secreted in a culture medium with copper excess (Cherrad et al., 2012) and BcXyn10A in media supplemented with tomato, strawberry pulp (Shah et al., 2009) or carboxymethylcellulose (Fernández-Acero et al., 2010). We have identified BcXyn11A as a component of the early secretome of *B. cinerea* grown in glucose and in tomato-fruit extract (Espino et al., 2010), which is consistent with the expression pattern described in this work, and, as far as we know, BcXyn11B has not been identified in any proteomic analysis. Here we show that the *Bcxyn11B* transcript is the least abundant in ungerminated conidia and, although this gene was the most upregulated in all growth conditions tested (**Figures 3**, **4**), the relative abundance of *Bcxyn11B*-mRNA still remained low compared with the other four xylanase transcripts. This could explain why BcXyn11B has not been detected yet.

The expression of the five xylanase coding genes was strongly enhanced by the fungus-host interaction (**Figure 5**). All genes, except *Bcxyn11C*, were upregulated very early after the germination of conidia in tomato leaves, and mRNA levels continued to increase as infection progressed. The expression of *Bcxyn11C* was only induced late in the infection (**Figure 5**). These results suggest a putative role of both GH10 xylanases, BcXyn11A and, to a lesser extent, BcXyn11B during both the early and late stages of the infective process, and of BcXyn11C in the maceration of the plant tissue, probably producing nutrients by hydrolysis of the plant hemicelluloses in the later stages of infection. In previous transcriptome analysis during *Botrytis*-plant interaction, different gene expression patterns have been observed. Only *Bcxyn11A* mRNA was detected after 96 h of *B. cinerea*-cucumber interaction, (Kong et al., 2015). Meanwhile, all xylanase coding genes, except *Bcxyn10A*, had been shown to be upregulated during colonization of sunflower cotyledons (Amselem et al., 2011) and all of them, but *Bcxyn11B*, had been reported to be induced in infected ripe tomato fruit, lettuce leaves and grape berries (Blanco-Ulate et al., 2014). However, so far, proteomic approaches have identified only BcXyn10A *in planta*, after 3 days of inoculation of *Botrytis* in tomato fruits (Shah et al., 2012). These results suggest that the induction pattern of each xylanase gene varies for different hosts.

In fungi, different transcription factors are involved in the regulation of glycosyl hydrolase genes (Amore et al., 2013; Glass et al., 2013) and the consensus binding motifs for some of them were searched in *B. cinerea* xylanase promoters (Supplementary Figure S3). Only the *Bcxyn11B* promoter showed a putative binding site for the *Aspergillus niger* xylanolytic activator XlnR (ortholog to *AoXlnR* in *Aspergillus oryzae* and *XYR1 in Trichoderma reesei*) (Noguchi et al., 2009; Amore et al., 2013). *Bcxyn11B* was the most upregulated in xylan (**Figure 2**), suggesting a potential effect by a putative XlnR-ortholog in *B. cinerea*. This finding points to the existence of both XlnR-dependent and XlnR-independent signaling pathways to control the expression of xylanase coding genes in *Botrytis*, as it has been described for *Aspergillus* species (Noguchi et al., 2009; Tani et al., 2012). Moreover, a potential recognition site for the global nitrogen regulatory protein Area, involved in regulation of a family of cellulases of *A. nidulans* (Lockington et al., 2002), was found in the promoter region of the *Bcxyn10B* gene (Supplementary Figure S3), suggesting the existence of an alternative regulation pathway for this gene by nitrogen metabolism. The five *B. cinerea* xylanase coding genes, but *Bcxyn11A*, might be regulated by pH, as the four promoters showed several putative binding sites for PacC (Supplementary Figure S3), the major factor involved in pH-dependent expression in *A. nidulans* (Peñalva and Arst, 2002). Finally, several potential recognition sites for the transcription factor CreA (Tudzynski et al., 2000) were predicted in all five xylanase promoters (Supplementary Figure S3). This transcription factor is functionally homologous to CreA of *A. nidulans*, which mediates the carbon catabolite repression of cellulase and hemicellulase genes and also represses the transcription of XlnR (de Vries et al., 1999). It has been shown that a functional CreA binding site requires two inverted repeat copies of the binding consensus sequence (Panozzo et al., 1998; Rauscher et al., 2006). However, no such sites were found in the xylanase-coding gene promoters (Supplementary Figure S3). This finding might explain the lack of repression by glucose detected for four of the five xylanase coding genes of *B. cinerea* (**Figure 3**).

Altogether, the results shown in this work and the previously reported secretome and transcriptome analysis point to a great diversity of signals that are able to regulate differentially the expression of the five endoxylanase coding genes in the *B. cinerea* genome.

### There Is a Cross-Talk between the Five *B. cinerea* Xylanases

The availability of a *Bcxyn11A* knockout mutant strain (Brito et al., 2006) allowed us to study whether the regulation of the remaining xylanases is different in this background. Depending on the growth conditions, the loss of BcXyn11A caused different alterations in the expression of the other four xylanases compared with the wild type strain (**Figure 6**). When the knockout mutant was grown in xylan as carbon source, or during its interaction with the plant tissue, the other two family GH11 xylanases were overexpressed in the mutant as compared with the wild type, but both GH10 xylanase coding genes showed reduced expression in the mutant (**Figure 6**). The use of tomato-fruit extract caused a different pattern of deregulation: *Bcxyn10B* and *Bcxyn11C* were repressed, but *Bcxyn10A* and *Bcxyn11B* were overexpressed (**Figure 6**). In the three conditions assayed, the mutation of *Bcxyn11A* induced the repression of the *Bcxyn10B* gene, suggesting that the products released by the enzymatic activity of BcXyn11A on its substrate might be necessary to induce the expression of *Bcxyn10B*. On the other hand, the overexpression of *Bcxyn11B* observed in Δ*Bcxyn11A* strain in all conditions tested, seems to be required to compensate the loss of BcXyn11A. Therefore, changes in the composition of the culture medium were able to induce additional modifications in the pool of xylanase transcripts, as observed during growth in tomato-fruit extract, to finally secrete an enzyme mix that can best counteract the lack of one of them, and that can hydrolyse better the various hemicelluloses present in each growth condition.

### The Efficiency of the Simultaneously Silencing of the Five Xylanases Coding Genes Depends of Growth Conditions

The nourseothricin and hygromycin selection systems have been the most widely used and the most efficient systems for *B. cinerea* genetic modifications, although recently the resistance to the fungicide fenhexamid has been proposed as a new selection marker (Cohrs et al., 2017). Therefore, the possibility to generate fungal strains with multiple gene deletions is so far limited to only 3 genes that could be manipulated in a single strain. In this work, we intended to use Post-Transcriptional Gene Silencing as an alternative approach for simultaneous silencing of the five *B. cinerea* endoxylanase coding genes. To do that, we designed the Hom_Xyl chimeric sequence, which contains 50-nt fragments from the xylanase genes that include those 21-nt regions from each gene with the maximum score to generate a specific siRNA, according with SVM RNAi server (Supplementary Figure S1). We obtained knockdown strains by transformation with the silencing vectors (Supplementary Figure S1) expressing constitutively the Homo_Xyl antisense mRNA (strain BcXyl-AS) or both sense and antisense transcripts (strains BcXyl-DT1 and BcXyl-DT2), and the putative downregulation of the five genes was assayed by Q-RT-PCR. The results showed a simultaneous downregulation of the five xylanase coding genes when the strains were grown in tomato-fruit extract medium (**Figure 7**), although the silencing degree varied between the five genes. As far as we know, this is the first example for *B. cinerea* silencing with gene fragments as small as 50 nt and of simultaneous downregulation of five different genes.

*Bcxyn11B* and *Bcxyn11C* genes escaped silencing in knockdown strains grown in xylan (**Figure 7**), while during the interaction with the host, three xylanase genes were successfully silenced but two were overexpressed (**Figure 7**), highlighting a connection between the silencing efficiency and the growth conditions. In accordance with this, similar results were found for the co-silencing of 10 xylanases in *Magnaporthe oryzae* using as silencing trigger a chimeric gene containing a small 40-nt region from each target gene (Nguyen et al., 2011). The growth of these *M. oryzae* knockdown strains in xylan caused the upregulation of some xylanase genes, indicating a loss of effectiveness of the silencing strategy (Nguyen et al., 2011). On the other hand, also in *M. oryzae*, the simultaneous silencing of nine cellulases belonging to glycoside hydrolase families 6 and 7 occurred during the vegetative growth of the knockdown strains, but not during infection of the plant tissues (van Vu et al., 2012). Finally, in *Cladosporium fulvum*, the fusion of the first exons of the six known hydrophobins was used as a silencing trigger and dissimilar levels of silencing for the target genes at different stages of the fungal life cycle were reported (Lacroix and Spanu, 2009). Therefore, the efficiency of RNA silencing of multiple members in a gene family seems to depend on the growth conditions, as we found for the simultaneous silencing of xylanase coding genes in *B. cinerea*.

The growth of *Botrytis* knockdown strains was not significantly different from the wild type strain (**Figure 8**). In xylan, the expression levels of *Bcxyn11B* and *Bcxyn11C* remained almost similar as in B05.10 strain, while the expression of the other 3 xylanase coding genes was significantly reduced (more than 95, 60, and 20% for *Bcxyn10B*, *Bcxyn11A* and *Bcxyn10A*, respectively) (**Figure 7**). These results suggest that at least BcXyn10B and BcXyn11A xylanases are not essential for the use of xylan as a nutrient, and that BcXyn11B and BcXyn11C might be the main enzymes responsible for the hydrolysis of this polysaccharide, at least in axenic culture. Similarly, in tomato-fruit extract (**Figure 8**), the co-silencing of the five xylanase coding genes did not affect the growth of the knockdown strains (**Figure 7**). Probably, the hemicellulose concentration in this culture medium was enough to induce the expression of the five genes (**Figure 3**), but not essential to drive the fungal growth. In any case, the degree of silencing was not sufficient to prevent the detection of xylanase activity in the culture medium for the knockdown strains (**Figure 8C**), although a clear reduction of the xylanase activity, higher than 40%, was observed for the three knockdown strains as compared with strain B05.10 (**Figure 8C**). Unexpectedly, the reduction in xylanase activity was similar for the Δ*Bcxyn11A* strain and the knockdown strains (**Figure 8C**). The deletion of *Bcxyn11A* gene caused a significant decrease of *Bcxyn11C* and *Bcxyn10B* transcript levels (80 and 56%, respectively) and an increase of the expression of *Bcxyn11B* and *Bcxyn10A* genes (1.4 and 2-fold, respectively) (**Figure 6**). These results suggest that the previous estimation that BcXyn11A was responsible for about one third of the endoxylanase activity produce by *B. cinerea* (Brito et al., 2006) might be an underestimation, since the lack of BcXyn11A actually caused the overexpression of other xylanases (**Figure 6**).

Finally, the putative role of endoxylanases in *B. cinerea* virulence could not be solved making use of the multiple genes silencing approach, as the expression of *Bcxyn11A* and *Bcxyn11C* genes, contrary to what was observed in axenic culture, escaped silencing and were overexpressed during the infection of tomato leaves with knockdown strains (**Figure 8**). Nevertheless, BcXyl-AS, but not BcXyl-DT strains, showed a 40% reduction of the infection expansion rate in tomato leaves compared with the wild type (**Figure 8D**). The degree of silencing of the five xylanase coding genes during the infection of tomato leaves was quite similar in the three knockdown strains (**Figure 7**), although a slight increase of the *Bcxyn10B* gene silencing could be observed for the BcXyl-AS strain (98% compared with 90% for the other two knockdown strains) (**Figure 7**), pointing to a putative role of BcXyn10B in the fungus-host interaction, although clearly further analysis are necessary to determine the role of xylanases in the pathogenesis of *B. cinerea*.

Overall, our results show that the expression of a chimeric gene containing regions as small as 50 nt of each target gene is sufficient to trigger RNA silencing of multiple genes in *B. cinerea*, although the efficiency of this strategy depends on the growth conditions and also on the possible cross-regulation of the expression by genes of the same family. This situation made difficult to use this approach to analyze the role of the endoxylanases in the fungal biology and pathogenesis, as some of these proteins escaped from silencing or were even overexpressed.

## Author Contributions

All authors participated in the design of the experiments as well as the analysis/evaluation of the results. NG and MG drafted the initial manuscript and all authors participated in the editing and approved its final version.

## Conflict of Interest Statement

The authors declare that the research was conducted in the absence of any commercial or financial relationships that could be construed as a potential conflict of interest.

## Footnotes

1 http://fungi.ensembl.org/Botrytis_cinerea

2 http://pfam.xfam.org

3 http://prosite.expasy.org

4 https://www.ebi.ac.uk/Tools/msa/clustalo/

5 https://www.ebi.ac.uk/Tools/psa/emboss_stretcher/

6 http://cbs.dtu.dk/services/SignalP

7 http://cbs.dtu.dk/services/NetNGlyc

8 http://cbs.dtu.dk/services/NetOGlyc/

9 http://web.expasy.org/protparam

10 http://changbioscience.com/stat/sirna
