# Supplementary material for: Simultaneous Silencing of Xylanase Genes in Botrytis cinerea
Source: Front Plant Sci. 2017 Dec 22;8:2174. doi: 10.3389/fpls.2017.02174 (PMC5743704; doi:10.3389/fpls.2017.02174)
Supplement: Supplementary file 1 [file Presentation_1.PDF]

Supplementary Material

Simultaneous silencing of xylanase genes in Botrytis cinerea

Néstor García, Mario González, Celedonio González and Nélida Brito*

*** Correspondence:** Dr. Nélida Brito; nbrito@ull.edu.es

Supplementary Tables

Supplementary Table S1. Oligonucleotides used in this study. Restriction sites are underlined.

Oligonucleotide	Sequence (5'→3')	Target gene	Purpose
XYL-FW(*Not*I+*Bam*HI)	CTACGATGCGGCCGCGACGATGGATCCAACCGCGACTTTTAACCAG	Hom_Xyl	pNAH-Xyl construction and genomic characterization of knockdown strains by PCR
XYL-RV(*Nco*I)	TAGCCATGGTTCGCTGGGGGTGGAGC	Hom_Xyl	
XYL-FW(*Nco*I)	ATCCCATGGAACCGCGACTTTTAACCAG	Hom_Xyl	pNDN-Xyl construction and genomic characterization of knockdown strains by PCR
XYL-RV(*Not*I+*Bam*HI)	CTACGATGCGGCCGCGACGATGGATCCTTCGCTGGGGGTGGAGCC	Hom_Xyl	
XYL-FW(*Not*I)	CTACGATGCGGCCGCAACCGCGACTTTTAACCAG	Hom_Xyl	pNDN-Xyl-Tail construction
LINK-RV(*Bam*HI)	CGATGGATCCTTAATTAAGGCCGGCCG	Hom_Xyl	
NiiA-B05-FW	AAGATACACGCATATCGG	*BcniiA* gene from *B. cinerea*	Genomic characterization of knockdown strains by PCR
NiiA-B05-RV	GATTTTTGACTTCTTGGC		
NiiA-FW-check	GGTGACAGTGAAGGATGC		
NR-FW-check	AGGATGGTTTGGTTTCGG	*BcniaD* gene from *B. cinerea*	Genomic characterization of knockdown strains by PCR
NR-MBD-BAM	CGGGATCCACCTCCTTTAAGCGCTTT		
NR-MBD-SMA	TCCCCCGGGTTTACCATGATCGGGTCGA		
NDN-RV	TAATAAACGCTCTTTTCTCTTAGGTTTACTTCACGACAATAGCACGGAC		
NOUR-RV-check	CAGGCGCTCTACATGAGC	*nat1* from *S. noursei*	Genomic characterization of knockdown strains by PCR
CHECK-RV2	GAATCCACTTACCTTCCG	*OliC* promoter from *A. nidulans*	Generation of the probe used in southern-blots
Hyg5R- Sal	GACGTCGACGAGCCGCATTCCCGATT		
NGH10_00576.1_FW	GGAAAGATCTATGCATGGGATG	Bcin03g03480 (*Bcxyn10A*)	Q-RT_PCR
NGH10_00576.1_RV2	AGGTTGCTGAATCCAGGTTG		
NGH10_01778.1_FW2	GGTACCTGGAGATCATTTGTCTTC	Bcin05g06020 (*Bcxyn10B*)	
NGH10_01778.1_RV	CTTGGAGAGACTTAACGAGGTTG		
NGH11_797_FW2	GTTGGTTCCGAGCGCTC	*Bcin03g00480* (*Bcxyn11A*)	
NGH11_797_RV	GCTTGAAGGTAGCAGTTCCTTG		
NGH11_13645.1_FW	GCTGGACAACTGGATCTACTAATCC	Bcin15g01600 (*Bcxyn11B*)	
NGH11_13645.1_RV	CTTGACCCGTCGCTTGTG		
NGH11_03590.1_FW	GAATCCTGGAAGTGCTAAGGC	Bcin12g00090 (*Bcxyn11C*)	
NGH11_03590.1_RV	TGCTCCGTCACTAACCACG		
ActA-FW	GGTAACATTGTTATGTCTGG	*actA* from *B. cinerea*	
ActA-RV	CTTGACCTTCATCGACG		

Supplementary Table S2. Protein query sequences used in the BLAST-P searches against the *Botrytis cinerea* genome database.

PDB[a] accession nº.	Uniprot[b] accession nº.	GH family[c]	Protein	Organism
1BK1	P33557	GH11	Endo-1,4-beta-xylanase C	*Aspergillus kawachii*
1XYN	P36218	GH11	Xylanase I	*Trichoderma reesei*
2DFB	P36217	GH11	Xylanase II	*Trichoderma reesei*
1TE1	Q9HFH0	GH11	Endo-1,4-beta-xylanase C	*Penicillium funiculosum*
3U7B	B3A0S5	GH10	Endo-1,4-beta-xylanase A	*Fusarium oxysporum*
4XUY	A2QFV7	GH10	Endo-1,4-beta-xylanase C	*Aspergillus niger*
3U7B	B3A0S5	GH10	Endo-1,4-beta-xylanase A	*Fusarium oxysporum*

[a] RCSB Protein Data Bank (PDB) website: http://www.rcsb.org/pdb/home/home.do
[b] Universal Protein Resource (UniProt) website: http://www.uniprot.org
[c] CAZy database glycosyl hydrolase (GH) classification: website http://www.cazy.org/

Supplementary Figures

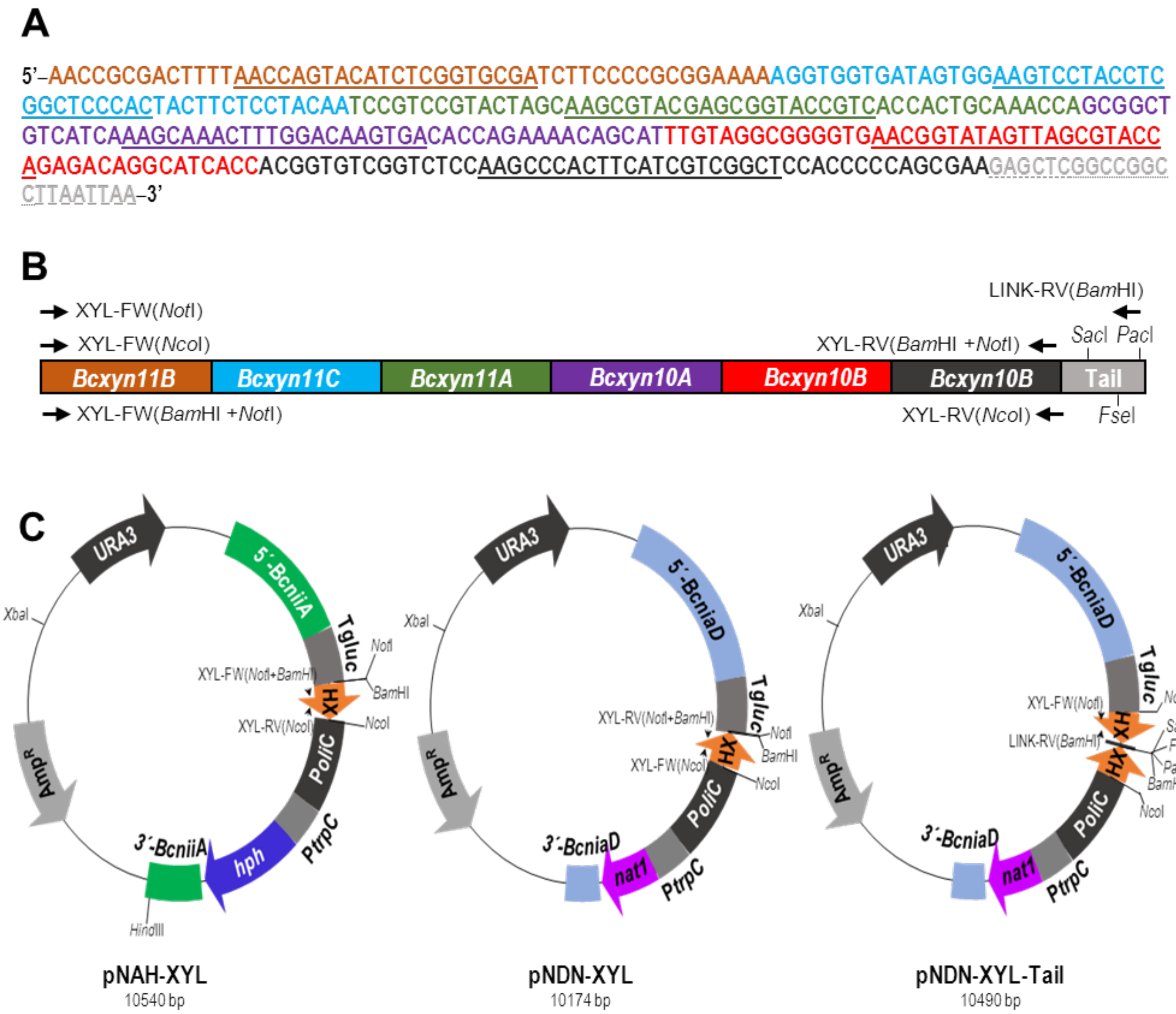

Supplementary Figure S1. The chimeric sequence Hom_Xyl and the silencing vectors. A) Sequence of the chimeric gene Hom_Xyl, in which the 50-nt region of each gene is denoted by distinct colors. Regions of 21 nt with the maximum score to generate specific siRNAs according to SVM RNAi server are underlined. It continues at the 3'-end with the 22 nt-tail (gray) and restriction sites are indicated by broken underlining. **B)** Simplified scheme of the Hom_Xyl fragment. Each 50-nt region is represented by a colored box as in A) and the primers used for Hom_Xyl amplification are shown as arrows. **C)** Plasmid maps of the silencing vectors generated in this work. HX, Hom_Xyl fragment; P*oliC*, *OliC* promoter from *A. nidulans;* T*glu*, glucanase terminator from *B. cinerea; 5'-BcniiA and 3'-BcniiA*, sequences upstream and downstream of the *B. cinerea* BcNiiA ORF, respectively; PtrpC, trpC promoter from *A. nidulans*; hph,, hygromycin B phosphotransferase gene from *E. coli*; *5'-BcniaD and 3'-BcniaD,* sequences upstream and downstream of the *B. cinerea* BcNiaD ORF, respectively; *nat1*; nourseothricin acetyl transferase1 gene from *Streptomyces noursei*.

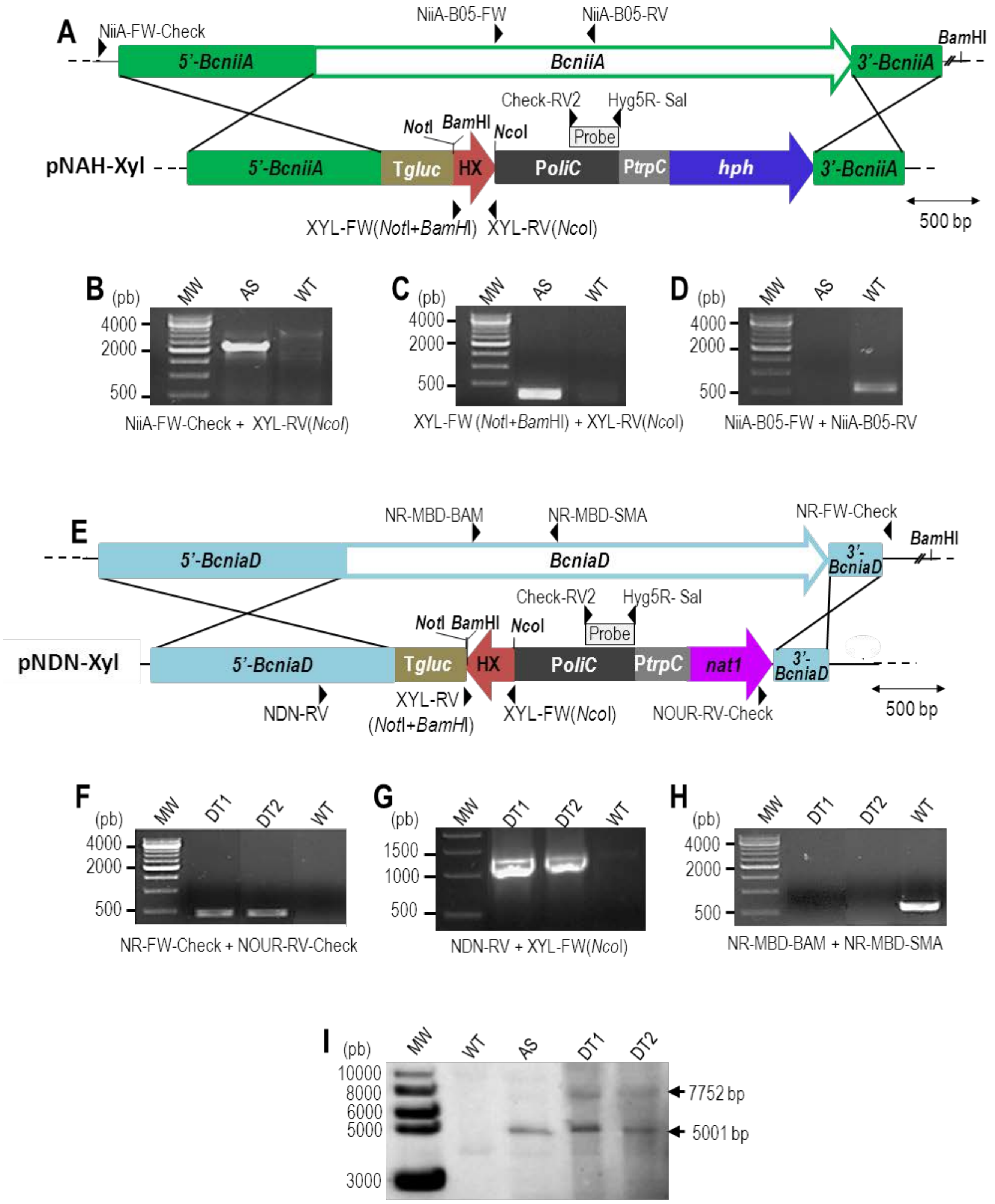
A
NiiA-FW-Check
NiiA-B05-FW
NiiA-B05-RV
5'-BcniiA
BcniiA
3'-BcniiA
BamHI
Check-RV2
Hyg5R- Sal
NotI
BamHI
NcoI
Probe
pNAH-Xyl
Tgluc
HX
PoliC
PtrpC
hph
XYL-FW(NotI+BamHI)
XYL-RV(NcoI)
500 bp
B
(pb)
MW
AS
WT
4000
2000
500
NiiA-FW-Check + XYL-RV(NcoI)
C
XYL-FW (NotI+BamHI) + XYL-RV(NcoI)
D
NiiA-B05-FW + NiiA-B05-RV
E
NR-MBD-BAM
NR-MBD-SMA
NR-FW-Check
5'-BcniaD
BcniaD
3'-BcniaD
pNDN-Xyl
nat1
NDN-RV
XYL-RV (NotI+BamHI)
XYL-FW(NcoI)
NOUR-RV-Check
F
DT1
DT2
NR-FW-Check + NOUR-RV-Check
G
1500
1000
NDN-RV + XYL-FW(NcoI)
H
NR-MBD-BAM + NR-MBD-SMA
I
10000
8000
6000
5000
3000
7752 bp
5001 bp

Supplementary Figure S2. Generation of knockdown strains for GH10 and GH11 xylanases from *Botrytis cinerea.* A) Schematic representation of the double homologous recombination even replacing the BcNiiA ORF in the *Botrytis* genome with the silencing construct contained in pNAH-Xyl plasmid, generating the BcXyl-AS knockdown strain. Primers used in PCR reactions carried out for the characterization of the transformants are shown. **B)** Agarose gel electrophoresis of amplicons obtained by PCR from genomic DNA of BcXyl-AS (AS) and B05.10 (WT) strains with NiiA-FW-check and XYL-RV(*Nco*I) primers. **C)** Idem as in B) with Xil-FW(*Not*I+*Bam*HI) and Xil-RV(*Nco*I) primers. **D)** Idem as in B) with NiiA-B05-FW and NiiA-B05-RV primers. **E)** Schematic representation of the double homologous recombination even replacing the BcNiaD ORF in the BcXyl-AS genome with the silencing construct contained in pNDN-Xyl plasmid, generating the BcXYL-DT1 and BcXyl-DT2 knockdown strains. The primers used in PCR reactions carried out for the characterization of the transformants are shown. **F)** Agarose gel electrophoresis of amplicons obtained by PCR from genomic DNA of BcXyl-DT1 (DT1), BcXyl-DT2 (DT2) and B05.10 (WT) strains with NR-FW-Check and NOUR-RV-Check primers. **G)** Idem as in F) with NDN-RV and XYL-FW(*Nco*I) primers; **H)** Idem as in F) with the NR-MBD-BAM and NR-MBD-SMA primers. **I)** Southern-blot of genomic DNA from the indicated strains digested with restriction enzyme (*Bam*HI) and hybridized with the probe shown in A) and E) and obtained by PCR with the indicated primers.

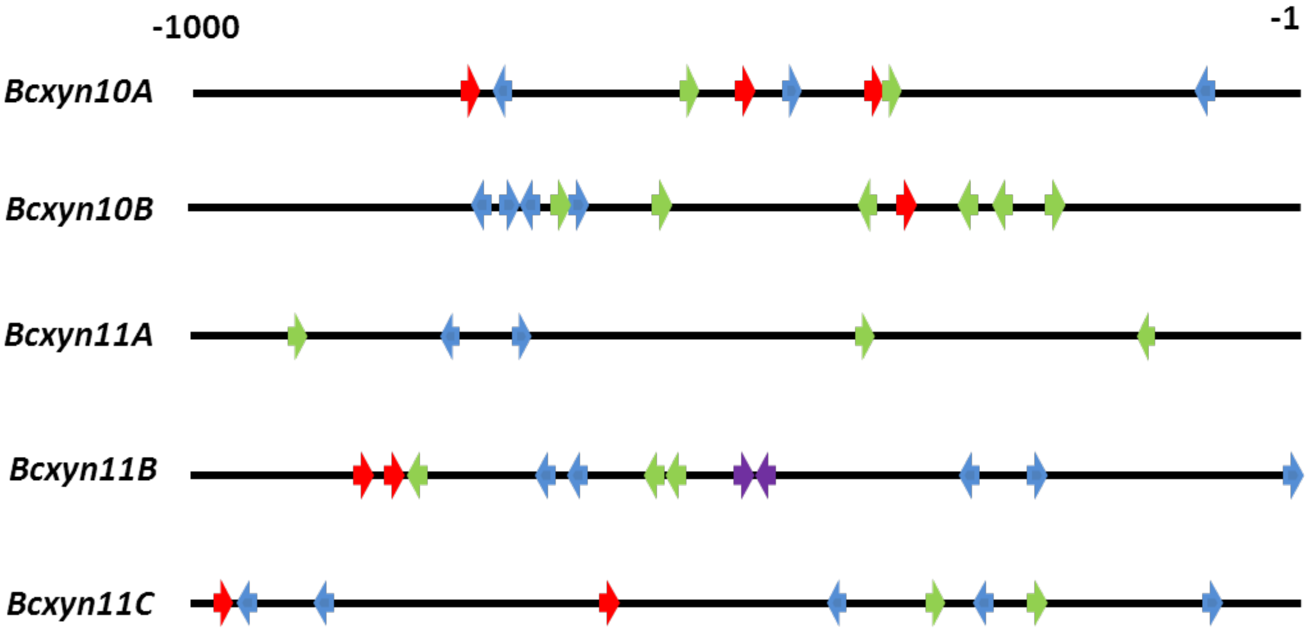

Supplementary Figure S3. Putative transcription binding sites in the promoter regions of xylanase coding genes of *B. cinerea*. For each gene, the promoter region 1000 bp upstream from the putative transcription start site is shown. The position of significant binding sites are indicated as arrows as follows: for the xylanolytic activator XlnR (Amore *et al.*, 2013), consensus sequence 5'-GGCTAAA-3'; for the major factor involved in pH-dependent expression PacC (Peñalva and Arst, 2002) consensus sequence 5'-GCCARG-3'; for the main regulator responsible for carbon repression, CreA (Tudzynski *et al.*, 2000), consensus sequence 5'-SYGGRG-3'; for the global nitrogen regulatory, Area, consensus sequence 5'- HGATAR-3'(Lockington *et al.*, 2002).

References

- Amore, A., Giacobbe, S., and Faraco, V. (2013). Regulation of cellulase and hemicellulase gene expression in fungi. *Curr. Genomics* 14, 230-249.
- Peñalva, M.A. and Arst, H.N., Jr. (2002). Regulation of gene expression by ambient pH in filamentous fungi and yeasts. *Microbiol. Mol. Biol. R.* 66, 426-446.
- Tudzynski, B., Liu, S. and Kelly, J.M. (2000). Carbon catabolite repression in plant pathogenic fungi: isolation and characterization of the *Gibberella fujikuroi* and *Botrytis cinerea creA* genes. *FEMS Microbiol. Lett.* 184, 9-15.
- Lockington, R.A., Rodbourn, L., Barnett, S., Carter, C.J. and Kelly, J.A. (2002). Regulation by carbon and nitrogen sources of a family of cellulases in *Aspergillus nidulans. Fungal Genet. Biol.* 37, 190-196.
